# Over-Selectivity is Related to Autism Quotient and Empathizing, But not to Systematizing

**DOI:** 10.1007/s10803-016-2990-3

**Published:** 2017-01-28

**Authors:** Phil Reed

**Affiliations:** Department of Psychology, Swansea University, Singleton Park, Swansea, SA2 8PP UK

**Keywords:** Over-selectivity, Autism quotient, Empathizing quotient, Systematizing quotient, Broad autistic phenotype

## Abstract

The relationships of autism quotient (AQ), systematizing (SQ), and empathizing (EQ), with over-selectivity were explored to assess whether over-selectivity is implicated in complex social skills, which has been assumed, but not experimentally examined. Eighty participants (aged 18–60) were trained on a simultaneous discrimination task (AB+CD−), and tested in extinction on the degree to which they had learned about both elements of the reinforced (AB) compound. Higher AQ and lower EQ scorers demonstrated greater over-selectivity, but there was no relationship between SQ and over-selectivity. These results imply that high AQ scorers perform similarly to individuals with ASD on this cognitive task, and that over-selectivity may be related to some complex social skills, like empathy.

## Introduction

The concept of a broad autistic phenotype implies that traits associated with autism spectrum disorder (ASD) are distributed throughout the population to vary degrees and with varying severities (e.g., Constantino and Todd 2003; Folstein and Rutter 1977; Plomin et al. 2009). For example, it has been suggested that close relatives of those with ASD will show some of the characteristics of those with ASD (e.g., Piven et al. 1997; Micali et al. 2004). Moreover, it has also been suggested that those scoring highly on psychometrically-defined measures of traits related to ASD should exhibit similar cognitive and behavioral characteristics to those demonstrated by individuals in clinical ASD samples (Baron-Cohen et al. 2001).

A number of different psychometric scales are commonly employed to assess the degree to which individuals might possess various autistic-like traits. The Autism Quotient (AQ) scale assesses individuals along a number of dimensions related to ASD: social skill, attention switching, attention to detail, communication, and imagination (Baron-Cohen et al. 2001). Relative to those with low psychometrically-defined autism traits (AQ), those who score highly on AQ have been shown to display greater self-focused attention (Lombardo et al. 2007), local rather than global processing (Grinter et al. 2009), have difficulty inferring others’ mental state from the eyes (Baron-Cohen et al. 2001) or attentional cueing from gaze (Bayliss and Tipper 2005), as well as narrowed visual search patterns (Reed et al. 2011). These findings of similarities between performance of those with high autism traits in nonclinical samples and those with clinically-defined ASD give support to the notion of a broad autistic phenotype.

One aim of the current study was to assess the degree to which AQ scores in a nonclinical population predict the existence of over-selective type responding by an individual. Over-selectivity refers to the phenomenon whereby behavior is controlled by one element of the environment at the expense of other equally salient stimuli (e.g., Leader et al. 2009; Lovaas et al. 1971; Reed et al. 2009). For example, understanding speech has been taken by some to involve not only understanding the sounds of the words but also interpreting the facial expressions that go with the words, and interference with the ability to attend to both inputs, such as is described by over-selective responding, can disrupt understanding speech (e.g., Jordan et al. 2000). It has been suggested that such over-selective responding could be implicated in a range of skills noted to be problematic for those with ASD, often involving social interactions of various kinds (Cumming and Berryman 1965; Lovaas et al. 1979; Schreibman and Lovaas 1973), but there has been very little experimental investigation of this assumed relationship.

Experimentally, over-selectivity has been studied using a simultaneous discrimination task in which participants initially are reinforced for selecting one compound stimulus (AB) in preference to another (CD). During a subsequent non-reinforced test, they are given a choice between individual elements of the previously reinforced stimulus and those from the previously non-reinforced compound (e.g., A v C, B v C, etc.). Participants displaying over-selectivity choose one element from the previously reinforced compound (e.g., A) in preference to elements from the previously non-reinforced compound (C and D), to a greater extent than they select the other previously reinforced element (e.g., B) in preference to C or D.

Although over-selectivity is a common problem for individuals with ASD (see Dube 2009; Ploog 2010, for reviews), its existence in those with high AQ scores has not been established, and this basic finding would extend the range over which the performance of individuals with high AQ is similar to that of those with ASD. Over-selectivity has been noted in a typically-developing population lacking any neurological damage, but tends to be seen more readily when an additional cognitive load is employed concurrently with the discrimination task (e.g., Reed and Gibson 2005; Reynolds and Reed 2011). The current study did not use such a load in order to see if higher AQ scores would be associated with greater over-selectivity in the absence of such a procedure designed to induce its presence.

However, the current study had further goals in addition to exploring this aspect of cognitive performance for high AQ scorers. In assessing the BAP, other scales have been developed to determine the degree to which individuals might display other cognitive styles associated with ASD; especially, systematizing (Baron-Cohen et al. 2003) and empathizing (Baron-Cohen and Wheelwright 2004). ‘Systemizing’ (measured by the systematizing quotient; SQ) reflects an individual’s drive to analyze the variables in a (usually inanimate) system, in order to understand the rules and mechanisms that govern that system; whereas ‘empathizing’ (measured by the empathizing quotient; EQ) assesses the degree to which individuals understand the emotions of others (Baron-Cohen and Wheelwright 2004). The latter two scales have been developed based on a particular view of sexual dimorphism in ASD, as involving a bias towards systems—often characterized as the ‘male brain’ (Baron-Cohen et al. 2002). The impact of these psychometrically-defined traits has not been widely explored in terms of their effect on the cognitive processing abilities that are typically associated with ASD—such as over-selective responding, which would extend knowledge about these scales.

It might be expected, to the extent that all of these scales are associated with the BAP, they may all be associated with performance on any number of cognitive tasks. However, while it is not unique in this regard, over-selective responding does allow a number of theoretical and practical implications of high scores on each of the scales separately to be unpacked. Initially, it was suggested that low EQ and high SQ scores combine to produce a cluster of symptoms typically seen in those with clinical ASD (see Wheelwright et al. 2006). As a consequence, it might be predicted that AQ should be strongly positively related to SQ and negatively related to EQ. If this is the case, then, to the extent that AQ scores are associated with over-selectivity, both high SQ and low EQ would also be related to over-selectivity. However, the evidence relating to the associations between AQ, EQ, and SQ, and as they relate to the broad autistic phenotype, is somewhat mixed (see Barbeau et al. 2009), and all three scales do not always predict performance on tasks known to be impacted in those with ASD (see Voracek and Dressler 2006). Given this, it is not certain that this simple theoretical prediction would be borne out experimentally.

In contrast to the above, it may be that only one, rather than both, of the SQ and EQ scales might predict over-selectivity, and the nature of these specific relationships with over-selectivity might help to illuminate the nature of these psychometric ASD-related constructs. As noted previously, over-selective responding has been linked with higher-order ASD problem behaviors, involving social interactions of various kinds (Cumming and Berryman 1965; Lovaas et al. 1979; Schreibman and Lovaas 1973) that extend beyond low-level cognitive deficits such as attention (Dube 2009) or retrieval (Leader et al. 2009). For example, the ability to attend to multiple stimuli is implicated in the formation of many complex social abilities (Cumming and Berryman 1965), understanding of speech (Jordan et al. 2000; Lovaas et al. 1979), and social interactions (Reed and Steed 2015; Schreibman and Lovaas 1973). To the extent that empathizing is related to these social abilities, and the presence of over-selectivity reduces these abilities, it might be that EQ but not SQ, which is typically thought to relate to inanimate systems, is related to over-selective responding (low EQ predicting high over-selectivity). In contrast, if over-selectivity relates purely to low-level cognition and processing of stimulus input, then it might relate to high-order social skills (assumed to be associated with EQ), but over-selectivity could be associated with SQ scores—the latter being a stronger index of the ability to integrate information about inanimate systems. This latter view actually suggests a number of possible relationships between SQ and over-selectivity that depend on how the abilities clustering under psychometrically-defined SQ are conceptualized. If these abilities require parallel processing of information to arrive at a mechanistic account of a system, then high SQ should predict less over-selectivity. However, if high SQ requires the ability to process and integrate information in series, then SQ may not be impacted by over-selectivity, which is associated with processing multiple sources of information simultaneously.

Given the above unexplored possibilities, the current study examined the relationships between AQ, SQ, and EQ and over-selectivity. This would help extend the range of tasks over which AQ has been explored, allow investigation of whether AQ, SQ, and EQ are all related to such tasks, which may shed light on whether over-selectivity might be implicated in complex social skills in addition to simple low-level processing, which has been assumed but not experimentally explored.

## Methods

### Participants

Eighty participants (30 female, 50 male) were recruited from the general public through advertisement. The study was advertised as an investigation into personality and learning. The participants were not paid, or given any reward, for their contribution. The sample had a mean age of 37.13 (±14.79, range = 19–60) years. G-power calculations suggested that for a medium effect size (*f* = 0.25), using a significance criterion of *p* < .05, in order to obtain 95% power, the size of the total sample should be 54. There were a number of exclusion criteria applied to the study. In the advert it was specified that that study was only recruiting volunteers who were between 18 and 60 years old, the upper criterion was adopted as it is known that age impacts over-selectivity (Kelly et al. 2015; McHugh and Reed 2007), and who had no history of psychiatric problems or developmental or intellectual issues. Only two volunteers reported a history of mental health problems, and they were excluded from the study. Individuals with an AQ score of above 32 were excluded (as it is possibly that they had clinical ASD), and those with an IQ of below 80 were also excluded, as IQ is also known to impact over-selectivity (see Kelly et al. 2015). No exclusions were made on these bases.

### Apparatus and Materials

Wechsler Abbreviated Scale of Intelligence (WASI, Sattler 1998) measures intellectual ability, and is suitable for ages 6 to 89 years. It comprises four subtests, two assessing language (vocabulary and similarities), and two performance measures (block design and matrix reasoning). Thus, the WASI generates two scores of abilities, verbal and performance scores, and a full score of intellectual functioning. Test reliability has been stated at 0.87–0.92.

Autistic Spectrum Quotient Questionnaire (AQ; Baron-Cohen et al. 2001) measures the level of autistic traits that an individual may possess. This questionnaire consists of 50 questions, with a score of 32 generally being suggested as indicating high functioning ASD. The test–retest reliability of the scale is 0.70 (Baron-Cohen et al. 2001), and the internal consistency (Cronbach alpha) of the AQ is 0.82 (Austin 2005). There are sub-scales to the AQ, however, there is some debate about the appropriate factor solution for the AQ, and the reliabilities of the sub-scales are uncertain (see Austin 2005; Hurst et al. 2007). Given these concerns, only the overall AQ score was employed.

Systemizing Quotient (SQ; Baron-Cohen et al. 2003) assess interest in systems across a range of different classes of system. It comprises of 60 questions, 40 assessing systemizing and 20 distractor (control) items. It produces a maximum score of 80, and has an internal reliability of 0.78 (Auyeung et al. 2009).

Empathy Quotient (EQ; Baron-Cohen and Wheelwright 2004) measures empathy, and comprises 40 items. It produces a maximum score of 80, and has an internal reliability of 0.93 (Auyeung et al. 2009).

**Fig. 1:**
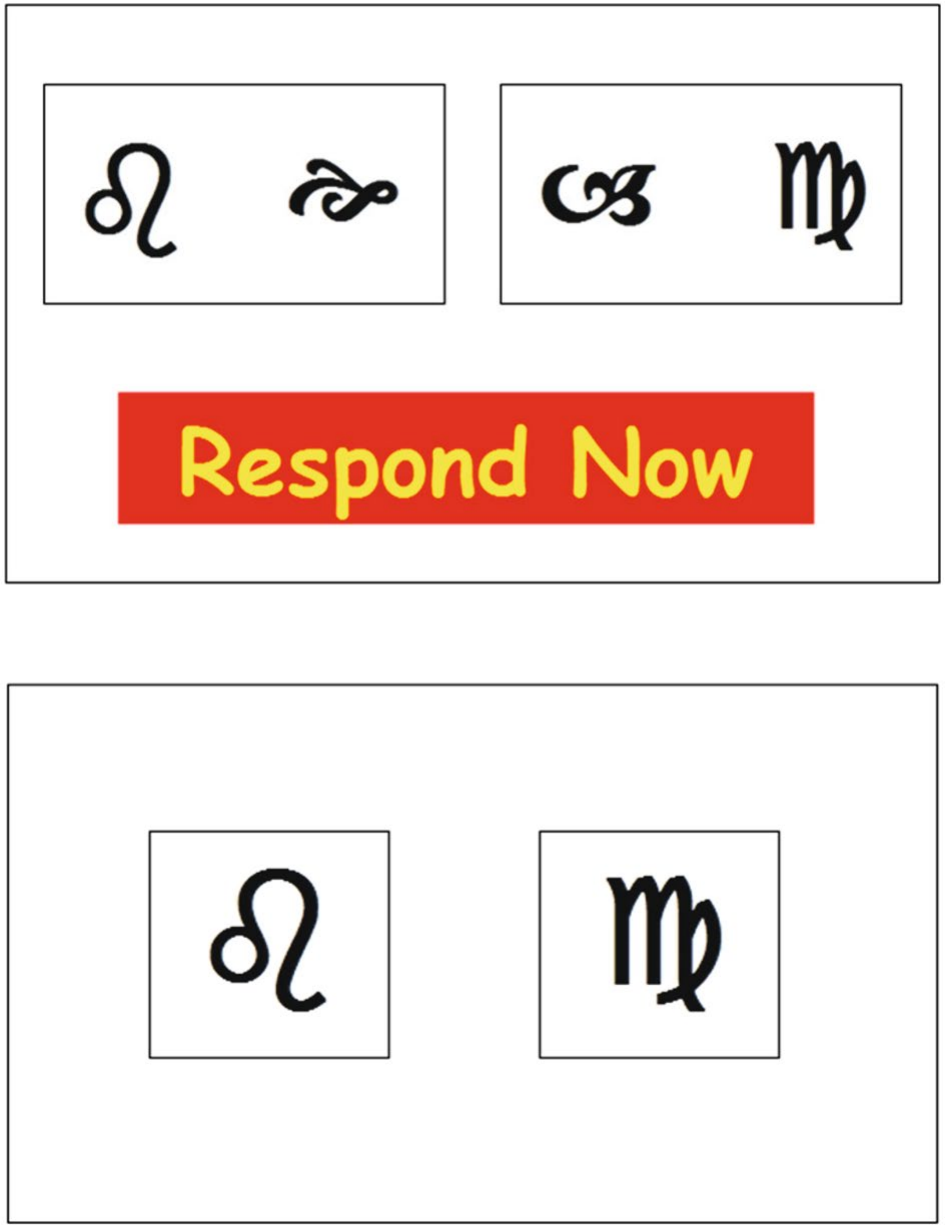
An example of the compound stimuli used during the training phase, followed by an example of the elemental stimuli used during the testing phase

Over-selectivity stimuli. Stimuli used during the procedure included eight abstract pictorial symbols taken from various fonts from Microsoft Word 2010 (Wingdings, Wingdings 2 and Symbol). Stimuli were either presented as a compound for training, or an elemental stimulus during testing. In all phases, each symbol appeared in black and measured approx. 5 cm × 5 cm (see Fig. 1).

### Procedure

All participants were tested individually in a small quiet laboratory cubicle containing a desk, a chair, and a computer. The over-selectivity procedure was automated on a Dell Latitude E6540 laptop (display size 15.5″).

*Training phase* Participants initially were presented with the instruction**s**: “Please select one of the two stimuli presented as soon as ‘respond now’ appears on the screen. You will be given feedback indicating whether you selected the correct or incorrect stimulus. Your aim is select the correct stimulus”.

All participants were then presented with a simple discrimination task consisting of the compound stimuli (AB vs CD). The compound stimulus AB appeared on the screen paired with compound stimulus CD; for half of the trials AB was on the right of the screen, and for the other half of the trials it was on the left (determined randomly). All participants received different symbols for each stimulus to control for the effects of intrinsic salience of the elements.

Participants selected one of the compounds when ‘Respond Now’ appeared on the screen by clicking the mouse cursor on one of the compounds. The instruction was presented 2 s after the stimuli were presented. If the participant selected the target compound stimulus (AB), then ‘Correct’ appeared on the screen; but if they selected the non-target compound (CD), then ‘Incorrect’ appeared on the screen. Feedback was presented immediately after a response, and the next trial commenced immediately. Thus, AB was always reinforced, and CD was never reinforced. If participants did not respond within 1.5 s, the next trial commenced. Training continued until the participant selected the correct compound consecutively ten times.

*Test Phase* After completing the training phase, the test phase instructions were presented. Participants were instructed: “Please select one of the two stimuli presented. The computer will not tell you whether you are correct or incorrect”. Participants were then presented with one stimulus from the previously reinforced compound (e.g., A or B) paired with a stimulus from the previously non-reinforced compound (e.g., C or D). Each combination (A vs C, A vs D, B vs C, B vs D) was presented five times; thus, there were 40 trials in total. Participants were required to select one of the cards using the mouse cursor. They were provided with no feedback and each trial appeared on the screen immediately after a response had been given.

Subsequent to completing the experiment, participants were asked to complete three questionnaires (AQ, SQ, EQ), and were given the WASI assessment.

## Results

Table 1 shows the mean (standard deviations) for the autism quotient (AQ), systematizing quotient (SQ), empathizing quotient (EQ), and IQ (WASI) scores, as well as the correlations between the variables [Pearson’s, except for those involving gender which were point biserial (positive = correlation with male)]. These data revealed a significant negative correlation between the autism quotient and empathy quotient, small positive correlations between being male and both autism quotient and systematizing quotient, a strong relationship between being female and the empathy quotient and a strong relationship between being older and the empathy quotient.

**Table 1:** Mean (standard deviations) for the autism quotient (AQ), systematizing quotient (SQ), empathizing quotient (EQ), and IQ (WASI) scores, as well as the correlations between the variables [Pearson’s, except for those involving gender which were point biserial (positive = correlation with male)]

		SQ	EQ	IQ		Gender	Age
AQ	13.24 (6.13)	0.164	−0.384***	0.056		0.280*	0.152
SQ	53.47 (17.25)		0.233*	0.019		0.262*	0.203
EQ	50.73 (11.73)			0.144		−0.537***	0.433***
IQ	100.47 (7.19)					−0.017	0.244*

*p < .05; **p < .01; ***p < .001

The sample was split at the mean for each of the three scales to create a lower and higher scoring group for each, as has been done for previous examinations of the impact of AQ on cognitive functioning (Grinter et al. 2009; Reed et al. 2011): AQ lower-scoring group (*n* = 48; mean = 9.10 ± 3.10; range = 2–13), and AQ higher-scoring group (*n* = 32; mean = 19.43 ± 3.89; range = 14–26); SQ lower-scoring group (*n* = 40; mean = 39.52 ± 7.24; range = 18–53), and SQ higher-scoring group (*n* = 40; mean = 67.42 ± 12.30; range = 54–114); EQ lower-scoring group (*n* = 47; mean = 42.40 ± 6.40; range = 22–52), and EQ higher-scoring group (*n* = 33; mean = 62.57 ± 5.87; range = 53–73).

**Fig. 2:**
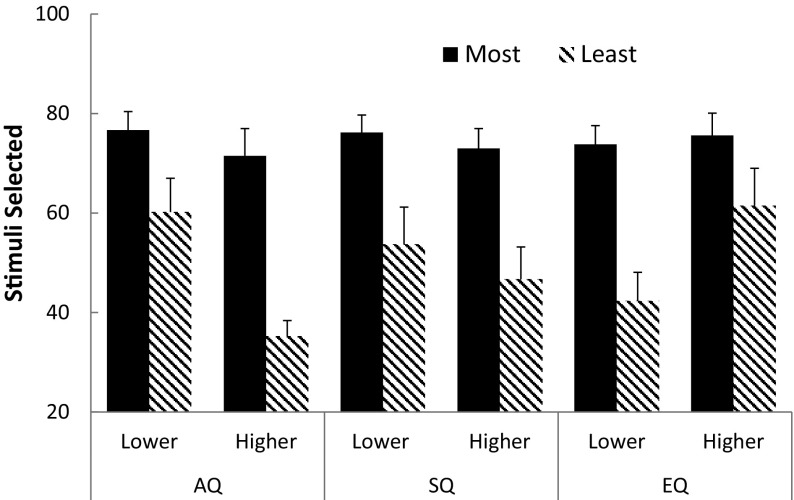
Number of stimuli chosen at test from previously reinforced stimulus, for lower and higher scoring autism (AQ), systematizing (SQ), and empathizing (EQ) groups. *Error bars* 95% confidence intervals

Figure 2 shows the results from the test phase of the experiment for both groups for each of the three scales. The percentage times that each element from the previously reinforced compound (AB) was chosen at test was calculated, and the percentage times that the most- and least-selected elements were chosen for each participant noted. Over-selectivity is indicated to the degree that one of the stimuli was chosen more often than the other at test. Inspection of Fig. 2 for the AQ scale reveals little difference between the most- and least-selected items for the lower-scoring group, but a large difference between the stimuli for the higher-scoring group. A two-factor mixed-model analysis of covariance (ANCOVA) with group (lower vs higher) as a between-subject factor, and stimulus (most vs least) as a within-subject factor, was conducted on these data; systematizing (SQ) and empathizing (EQ), IQ, as well as age and gender, were employed as covariates. In addition, the effect size (and its 95% confidence limits) was computed, as well as the Bayes factor for the null hypothesis (BF_0_) and the probabilities of the hypothesis (null and alternate) being true given the obtained data. The latter statistics were employed to determine whether any conclusions that depended on a null result were likely due to power issues. This analysis revealed significant main effects of stimulus, *F*(1,73) = 5.41, *p* < .05, *η* ^*2*^_*p*_ = 0.069 [95% CI = 0.069: 0.199]; *BF*_*0*_ = 0.513, *p*(*H*_*o*_/*D*) = 0.339, *p*(*H*_*1*_/*D*) = 0.661, and group, *F*(1,73) = 10.95, *p* < .001; *η* ^*2*^_*p*_= 0.130 [0.021–0.275]; *BF*_*0*_ = 0.033, *p*(*H*_*o*_/*D*) = 0.032, *p(H*_*1*_/*D)* = 0.976, and a significant interaction between the factors, *F*(1,73) = 7.95, *p* < .01; *η* ^*2*^_*p*_ = 0.098 [0.008–0.237]; *BF*_*0*_ = 0.143, *p*(*H*_*o*_/*D*) = 0.125, *p*(*H*_*1*_/*D*) = 0.874. Simple effect analyses between the two stimuli for the lower-scoring group revealed no significant difference between the stimuli, *F* < 1; *η* ^*2*^_*p*_ = 0.001 [0.000–0.003]; *BF*_*0*_ = 5.51, *p*(*H*_*o*_/*D*) = 0.846, *p*(*H*_*1*_/*D*) = 0.153, but a significant difference between the stimuli for the higher-scoring group, *F*(1,73) = 7.42, *p* < .01; *η* ^*2*^_*p*_= 0.092 [0.006–0.229]; *BF*_*0*_ = 0.184, *p*(*H*_*o*_/*D*) = 0.156, *p*(*H*_*1*_/*D*) = 0.844.

Inspection of these data for the SQ scale again reveals a difference between the most- and least-selected items at test for both lower- and higher-scoring groups. A two-factor mixed-model ANCOVA (group × stimulus; with AQ, EQ, IQ, age, and gender as covariates) revealed a significant main effect of stimulus, *F*(1,73) = 5.57, *p* < .05; *η* ^*2*^_*p*_  =0.071 [0.001–0.201]; *BF*_*0*_ = 0.471, *p*(*H*_*o*_/*D*) = 0.321, *p*(*H*_*1*_/*D*) = 0.679, but there was no significant main effect of group, *F* < 1; *η* ^*2*^_*p*_  = 0.002 [0.000–0.062]; *BF*_*0*_ = 8.38, *p*(*H*_*o*_/*D*) = 0.893, *p*(*H*_*1*_/*D*) = 0.106, or interaction, *F*(1,73) = 1.03, *p* > 0.30; *η* ^*2*^_*p*_= 0.014 [0.000–0.106]; *BF*_*0*_ = 5.06, *p*(*H*_*o*_/*D*) = 0.835, *p*(*H*_*1*_/*D*) = 0.165.

Inspection of these data for the EQ scale reveals that the difference between the most- and least-selected items for the lower-scoring group was greater than that for the higher-scoring group. A two-factor mixed-model ANCOVA (group x stimulus; with AQ, SQ, IQ, age, and gender as covariates) revealed no significant main effects of stimulus, *F*(1,73) = 2.50, *p* > .10; *η* ^*2*^_*p* =_ 0.033 [0.000–0.144]; *BF*_*0*_ = 2.32, *p*(*H*_*o*_/*D*) = 0.699, *p*(*H*_*1*_/*D*) = 0.300, or group, *F* < 1, *η* ^*2*^_*p*_ = 0.009, *η* ^*2*^_*p*_ = 0.008 [0.000–0.092]; *BF*_*0*_ = 6.30, *p*(*H*_*o*_/*D*) = 0.863, *p*(*H*_*1*_/*D*) = 0.137, but the interaction between the factors was significant, *F*(1,73) = 5.48, *p* < .05; *η* ^*2*^_*p*_ = 0.070 [0.001–0.200]; *BF*_*0*_ = 0.494, *p*(*H*_*o*_/*D*) = 0.330, *p*(*H*_*1*_/*D*) = 0.669. Simple effect analyses revealed a significant difference between the stimuli for the lower-scoring empathy group, *F*(1,73) = 4.89, p < .05; *η* ^*2*^_*p*_= 0.107 [0.000–0.147]; *BF*_*0*_ = 0.343, *p*(*H*_*o*_/*D*) = 0.255, *p*(*H*_*1*_/*D*) = 0.744, but no significant difference between the stimuli for the higher-scoring group, *F* < 1; *η* ^*2*^_*p*_ = 0.001 [0.000–0.074]; *BF*_*0*_ = 6.47, *p*(*H*_*o*_/*D*) = 0.866, *p*(*H*_*1*_/*D*) = 0.133.

A multiple regression was performed to see if any of the potential predictors (AQ, SQ, EQ, IQ, age, and gender) were related to the level of over-selectivity, as measured by the difference between the most- and least-selected stimuli at test. This analysis revealed a significant overall model, *F*(6,73) = 4.86, *p* < .001, *R* ^*2*^ = 0.285, with AQ (*β*= 0.134, *p* < .01) and EQ (*β* = −0.066, *p* < .05) being the only independently significant predictors of the difference (gender *β* = −0.888, *p* > .10; age *β* = −0.005, *p* > .70; SQ *β* = 0.006, *p* > .70; and IQ *β* = −0.027, *p* > .40).

In addition, a logistic regression was performed to determine if any of the values (AQ, SQ, EQ, age or gender) predicted over-selectivity. In the absence of any a priori method of determining the level of difference between the most- and least-selected stimuli at test needed for over-selectivity, the procedure recommended by Reynolds and Reed (2011) was adopted. The mean probability of choosing A and B was first calculated. Given this probability, the binomial equation was used to obtain the probability of choosing all possible combinations of A and B over C or D on ten trials. The probability of choosing a reinforced compound stimulus was set at the mean probability of choosing A and B stimuli in a particular condition. Then, the probability of obtaining 10 A, and zero to 10 B; the probability of obtaining 9 A, and 0–10 B; etc., were calculated, and put in a 10 × 10 contingency table. The contents of this table were then multiplied by a 10 × 10 table that contained the absolute A minus B difference score for each combination. The resulting 10 × 10 table contained the expected frequency of obtaining each possible A minus B difference resulting from all possible combinations of A and B frequencies. The sum of the values in this table (multiplied by 10) provided an estimate of the most minus least selected difference, in percentage terms, expected by random variation of selection of A and B stimuli. This gave a critical value of 21.3% difference between the stimuli to show over-selective responding (rounded to 30%). The overall regression produced a significant result, *X* ^2^(5) = 23.50, *p* < .001, −2*LL* = 86.15, Nagelkerke *R* ^2^ = 0.341. In terms of the predictors, this analysis revealed that AQ (odds ratio = 1.19, *p* < .01), and EQ (odds ratio = 0.978, *p* < .05) were significant predictors of over-selectivity (SQ odds ratio = 1.010, *p* > .60; IQ odds ratio = 0.948, *p* > .10; age odds ratio = 0.985, *p* > .40; gender odds ratio = 0.355, *p* > .10).

## Discussion

The current study demonstrated that an individual’s AQ score was associated with over-selective responding; those with higher AQ demonstrating greater over-selectivity. This finding has been shown for individuals with clinical ASD in comparison to typically-developing individuals (e.g., Leader et al. 2009; Reed et al. 2009), but not for those with high AQ scores. It should also be noted that, while the over-selectivity effect has previously been observed in typically-developing individuals (Reed and Gibson 2005; Reynolds and Reed 2011), this has only occurred when there has been a concurrent cognitive load task. The current study found the effect in high-scoring AQ individuals without such a cognitive load, and suggests that over-selective responding does not have to be induced in this population. This finding adds to the literature that suggests that those with high-scoring AQ show a similar cognitive style to those with ASD (e.g., Lombardo et al. 2007; Reed et al. 2011).

The results also demonstrated that an individual’s score on the EQ scale was related to the level of over-selectivity that they demonstrated—those with low EQ showed greater over-selectivity. This corroborates what has been suggested by a number of authors; namely, that complex social skills, such as empathy, require the processing of multiple stimuli, and that individuals who do not display strong abilities in these areas may also show over-selective responding (Cumming and Berryman 1965; Lovaas et al. 1979; Reed and Steed 2015). It has been previously suggested that situations in which over-selective responding might be seen are those in which individuals with ASD display impaired abilities, such as understanding speech (Jordan et al. 2000) or facial emotion recognition (Reed and Steed 2015), but there have previously been no demonstrations of a direct relationship between any higher-order social ability, such as empathizing, and over-selectivity.

However, there was no suggestion that systematizing (SQ) was associated with over-selective responding. Although it was the case that there were numerical differences in the over-selective responding (i.e. those with higher SQ showed more over-selective responding), this difference did not reach statistical significance, nor was this factor significant in any regression analysis. It should be noted that the power of the current tests were sufficient to produce a difference, and the Bayes statistics calculated suggested that was extremely unlikely that these differences were reliable. These findings also support the view that SQ and EQ are separable traits, and do not always predict performance together (e.g., Grove et al. 2013). There are a number of possibilities regarding the lack of association between SQ and over-selectivity in the current study. It may be that systematizing tendencies were not engaged for the current task, as it has been suggested that, while empathizing is automatic, systematizing is a controlled process only required in certain situations (Brosnan et al. 2014). Alternatively, it may be that systematizing reflects ability to process information in series, rather than in parallel. If this were the case, then it would not be necessary to attend to two cues at once, and SQ would not be related to over-selectivity. Clearly further studies are required to unpack these possibilities.

The other aspects of the current data support the view that there are some sex differences in relation to AQ, SQ, and EQ (Baron-Cohen et al. 2002; Wheelwright et al. 2006), but that only the latter (EQ) scale produced very strong differences in this regard in the current study. This finding is in line with the results reported by Wheelwright et al. (2006), who also noted larger sized effects for EQ compared to the other scales when comparing males and females. Also of note in the current data was the strong relationship between age and empathizing quotient, which has not previously been noted. This latter finding may depend on the way in which empathy is measured as Eysenck et al. (1985) noted no such relationship between empathy and age in their study using the Eysenck Personality Questionnaire. It may also be noted that it is unclear whether this relationship would hold if older individuals were included in the study. The current experiment excluded those aged over 60, due to concerns that age in itself can predict over-selective responding in older individuals (>70 years; e.g., Kelly et al. 2015). Further research might explore this older population as it might be that a bitonic relationship between EQ and age emerges if the age range is extended—this would certainly be predicted if over-selectivity and empathizing are related, as older people have been shown to shoe more over-selective responding (McHugh and Reed 2007; Kelly et al. 2015).

In addition to the specific relationships between over-selectivity and the various questionnaires employed in the current study, these results also have some implications for a number of theories of ASD that might be further discussed and explored. Of course, the current data was focused on exploring effects in the BAP, which may or may not be replicated within a clinical ASD population. This means that any such extrapolation should be made with caution and with the support of additional empirical evidence. Notwithstanding this proviso, the current data seems to bear on two theoretical views of ASD. Over-selective responding is clearly predicted from views of ASD such as weak central coherence (Happé and Frith 2006). However, it is unclear that such a view would predict the lack of relationship between systematizing and over-selective responding. According to the weak central coherence view, the same mechanisms are responsible for performance deficits in some situations and advantages in others. If enhanced systematizing is such a ‘double-edged’ mechanism that is tied to weak central coherence, then this view might have predicted high SQ scores would be related to over-selective responding. On the other hand, the theory of mind view of ASD (Baron-Cohen et al. 1985) may fare batter with these data, although the challenge for this view is why over-selectivity occurs across a range of non-social situations.

It is important to note that, although the relationship between over-selectivity and EQ suggests a role for the former in the disruption of empathizing, the use of one self-report measure of empathy (i.e., the EQ) does not answer the question of whether over-selectivity is implicated in other complex social skills (although see Cumming and Berryman 1965; Lovaas et al. 1979; Reed and Steed 2015; Schreibman and Lovaas 1973). Further studies using multiple tools, assessing multiple complex social skill domains, are needed to fully address the relationship between these skills and over-selectivity and the three psychometric scales employed. Indeed, such complex social skills may not be captured fully by any one or set of questionnaires, and ecologically valid studies might be usefully conducted to further this link to everyday social functioning. In addition, a limitation of the current study was that the sample was not specifically screened for psychiatric or neurological problems, other than by their own self-report. Although this is a common procedure, it may be useful to use a wider battery of tests in the future in regards to this issue. Whether over-selectivity is implicated in problems with social skills is an important question, particularly for informing interventions for those with ASD, and further work extending the current findings will be needed for a fuller answer to this question.

In summary, these results imply that those with higher psychometrically-defined AQ scores perform similarly to individuals with clinical ASD on this over-selectivity task. Furthermore, they give the suggestion that the relationship between over-selectivity and complex social skills, such as empathizing, may be an important one that could open a potential fruitful line of further study.
